# Gonococcal Infective Endocarditis: The Importance of a Sexual History

**DOI:** 10.7759/cureus.105434

**Published:** 2026-03-18

**Authors:** Shan Zaidi, Andrew Robinson, Danielle Zambrano, Peter Ebeid, Satyajeet Roy

**Affiliations:** 1 Medicine, Cooper Medical School of Rowan University, Camden, USA; 2 Infectious Disease, Mount Sinai Hospital, New York, USA; 3 Infectious Disease, Cooper University Hospital, Camden, USA; 4 Internal Medicine, Cooper Medical School of Rowan University, Camden, USA

**Keywords:** broad-range pcr, culture-negative infective endocarditis, neisseria gonorrhoeae, septic emboli, sexual history

## Abstract

*Neisseria gonorrhoeae* (NG), a commonly encountered sexually transmitted pathogen, rarely presents with cardiac involvement and therefore may be underrecognized in this context. Gonococcal infective endocarditis (IE) is associated with an aggressive clinical course characterized by rapid valvular destruction and systemic embolization. Diagnosis is often challenging, particularly when standard blood cultures fail to isolate the causative organism.

A 53-year-old man was admitted following a ventricular fibrillation arrest associated with ST-segment elevation and an acute headache. Magnetic resonance imaging demonstrated multiple acute and subacute cerebral infarcts, and chest radiography showed scattered reticulonodular opacities concerning for septic emboli. Transthoracic echocardiogram identified a mitral valve vegetation concerning for IE. Despite persistently negative blood cultures, the extent of valvular involvement prompted empiric antimicrobial therapy and eventual surgical valve replacement. Molecular testing performed directly on the valvular tissue detected NG, establishing the microbiologic diagnosis. Additional history later obtained during hospitalization disclosed high-risk sexual exposure without routine screening practices.

In modern clinical practice, NG IE is infrequently encountered yet continues to represent a severe form of disseminated infection characterized by extensive valvular damage, embolic sequelae, and mortality. NG's antigenic variability and evolving antibiotic resistance patterns may delay timely identification and complicate management, particularly in cases of culture-negative IE. Ceftriaxone remains the cornerstone of therapy; however, extensive valvular damage often necessitates surgical intervention. This case underscores the importance of a comprehensive sexual history and highlights the utility of universal polymerase chain reaction (PCR) when standard diagnostic testing does not establish an etiology.

In cases of destructive valvular involvement with embolic complications and persistently negative blood cultures, uncommon etiologies such as NG should remain within the differential diagnosis. Utilization of universal PCR may provide definitive pathogen identification and support appropriate therapeutic decision-making in this life-threatening condition.

## Introduction

*Neisseria gonorrhoeae* (NG) is a gram-negative diplococcus and one of the most common bacterial causes of sexually transmitted infections worldwide with an estimated global incidence of 86.9 million adults [1]. Classically, NG presents with urogenital symptoms and can also lead to a wide array of systemic complications when untreated or inadequately treated [2]. A severe manifestation of NG, disseminated gonococcal infection (DGI), most commonly presents with dermatitis, tenosynovitis, and migratory polyarthritis [2]. NG-associated rare invasive complications may occur and thus can create significant diagnostic and therapeutic challenges for clinicians.

NG infective endocarditis (IE), a highly virulent complication, is exceptionally rare with 1-2% of individuals with DGI developing it [3]. NG IE is associated with rapid valvular destruction, large vegetations, abscess formation, fistula development, and septic embolization [4]. Due to the extensive valvular damage, 72% of individuals will require surgical intervention [5]. Historically, in the pre-antibiotic era, NG was a more frequent cause of endocarditis; however, contemporary cases continue to be reported, often in younger patients without traditional risk factors for endocarditis [3,6]. Diagnosis may be challenging because NG is a fastidious organism that can be difficult to recover in routine blood cultures. Sodium polyanetholesulfonate (SPS), an anticoagulant commonly used in blood culture media, has been shown to be bactericidal to many gonococcal strains, leading to delayed growth or failure of recovery in a substantial proportion of isolates [7]. In addition, NG requires specialized growth media and carbon dioxide-enriched environments that are not consistently met in standard blood culture systems [2]. Due to the aggressive clinical course and potential for significant mortality, the importance of early recognition and management of NG IE is paramount, especially in patients presenting with systemic or atypical manifestations of NG infection.

This case was presented as a meeting abstract and poster at the 11th Annual Camden Scholars' Forum on May 2, 2024.

## Case presentation

A 53-year-old man with a relevant history of alcohol use disorder, hepatic steatosis, and tobacco use disorder presented with cardiac arrest due to ventricular fibrillation in the setting of an ST-segment elevation myocardial infarction. Additionally, he presented with an acute headache. His physical examination was remarkable for a systolic murmur heard in the mitral area. Initial laboratory evaluation demonstrated leukocytosis with a white blood cell count of 21.36×10⁹/L with neutrophilic predominance (86%; absolute neutrophil count 19.44×10⁹/L). Hemoglobin was 11.1 g/dL, and platelet count was 176×10⁹/L. Given his neurological symptoms, a computed tomography scan of the head was performed which was unrevealing.

**Figure 1:**
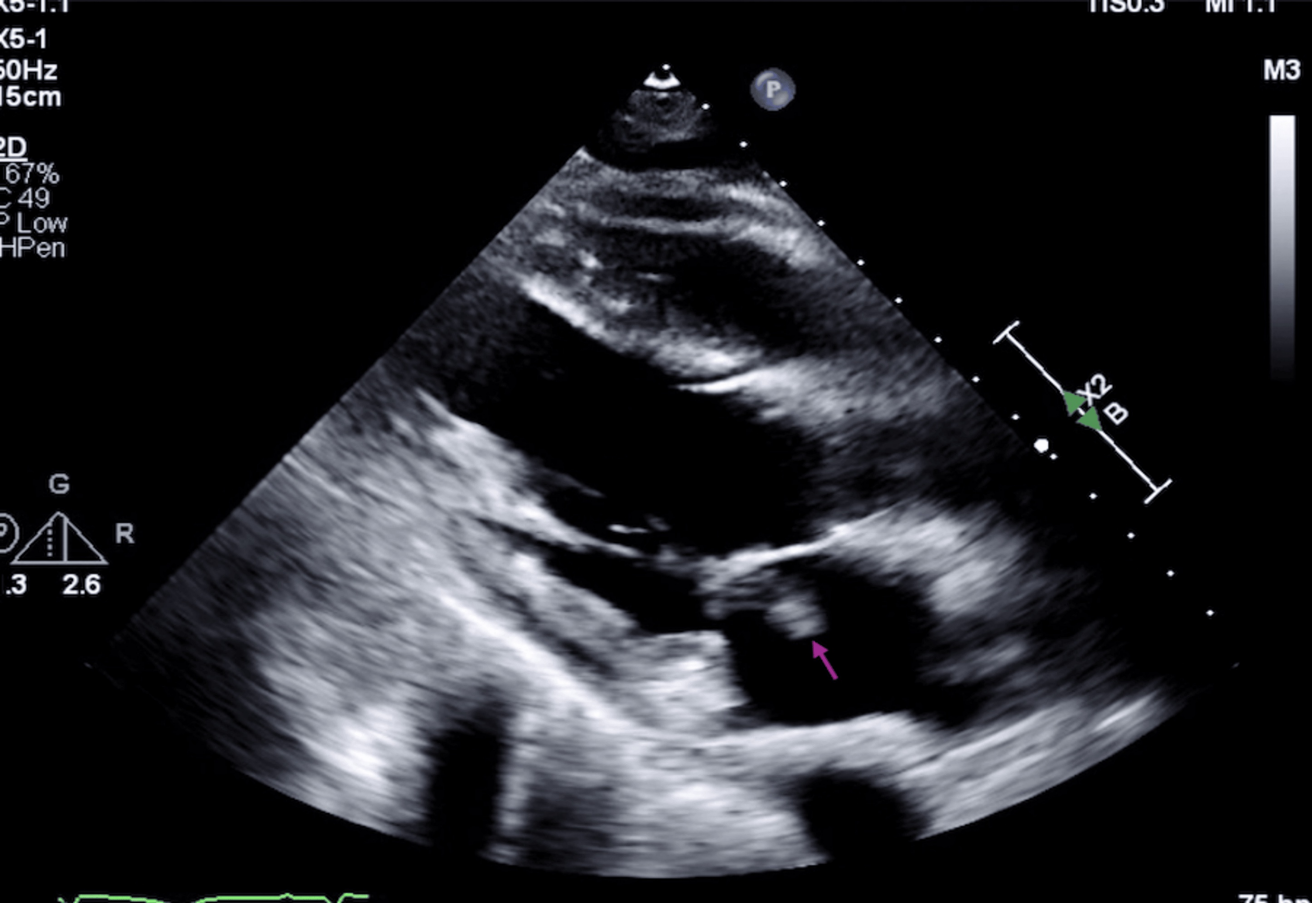
Parasternal long-axis transthoracic echocardiographic view demonstrating a large mitral valve vegetation (pink arrow)

**Figure 2:**
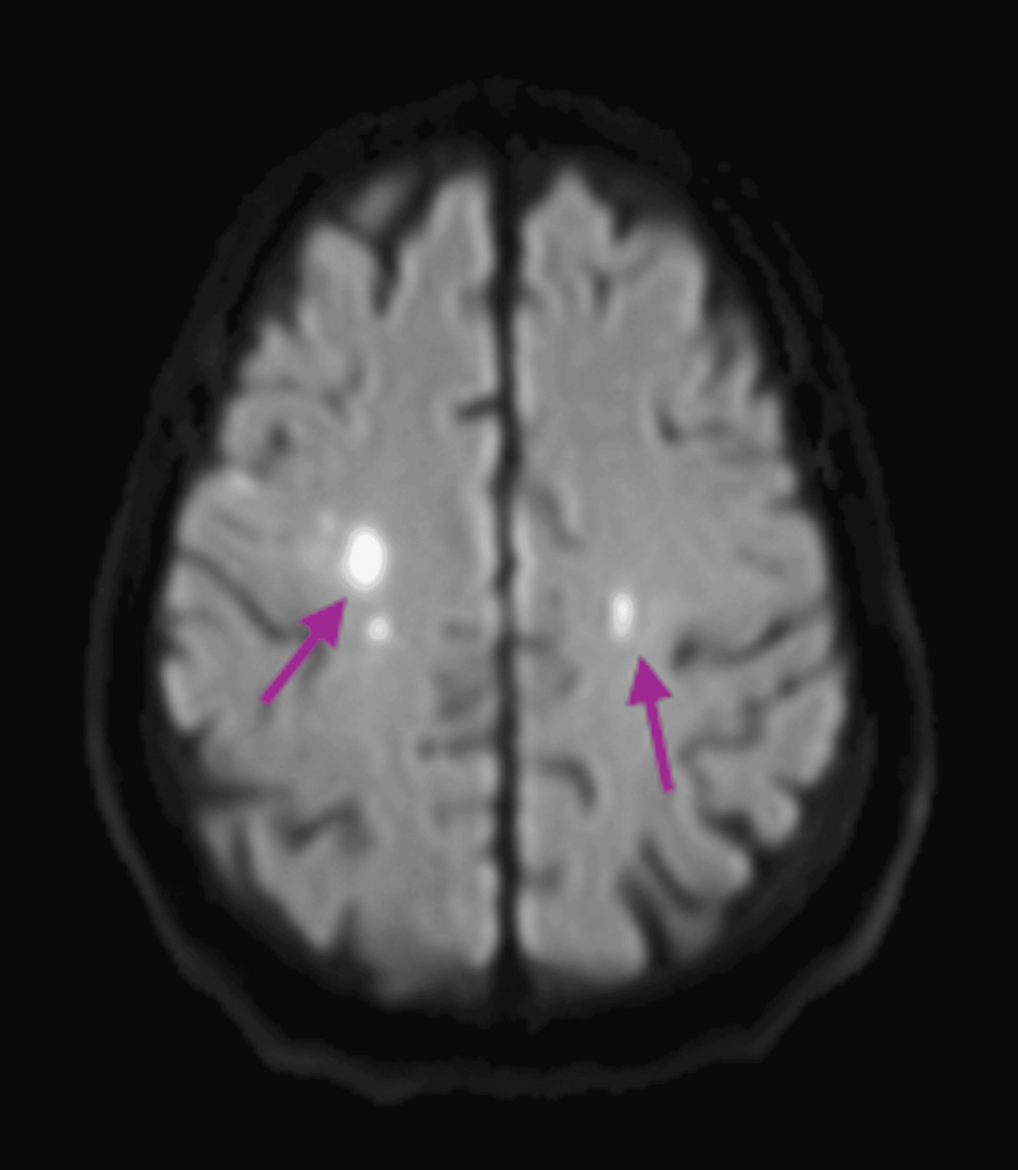
MRI of the septic emboli Axial diffusion-weighted imaging MRI sequence depicts small foci of restricted diffusion in the bilateral cerebral hemispheres in varying vascular distributions, most consistent with an embolic etiology. MRI: magnetic resonance imaging

**Figure 3:**
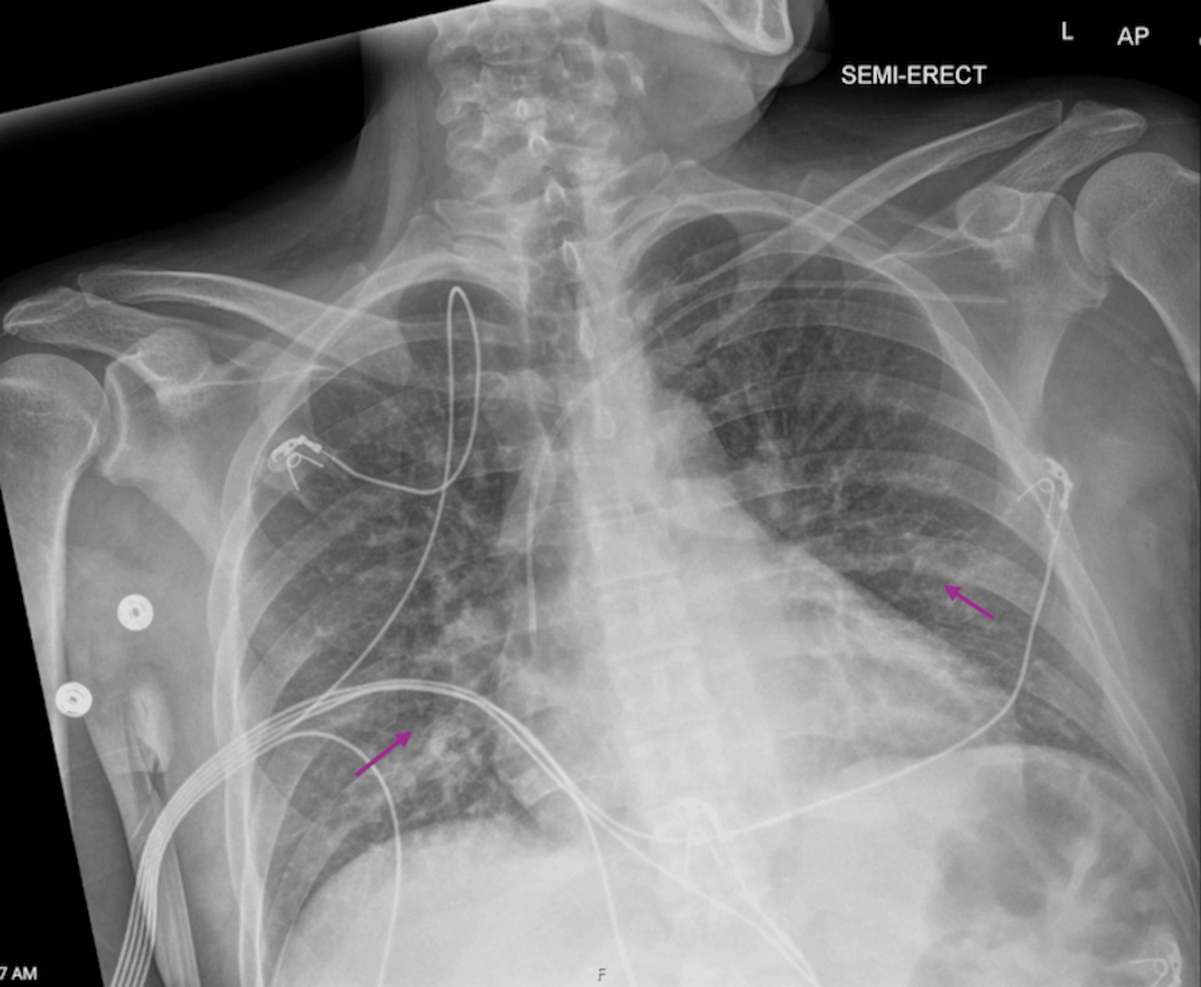
Chest radiograph demonstrating bilateral reticulonodular opacities concerning for septic pulmonary emboli

Further evaluation revealed evidence of systemic emboli. A transthoracic echocardiogram demonstrated a large mitral valve vegetation measuring 2.5×1.0 cm (Figure 1). Subsequent magnetic resonance imaging (MRI) of the brain revealed multiple acute versus subacute infarcts (Figure 2), and a chest X-ray showed scattered reticulonodular opacities concerning for septic emboli (Figure 3). His initial blood cultures were negative, and he was empirically treated with ceftriaxone and vancomycin for suspected IE.

Given the extensive vegetation and evidence of septic emboli, the patient underwent mitral valve replacement. On postoperative day 2, the patient developed bleeding complicated by a pericardial hematoma requiring emergent sternal re-exploration with ligation of the sinoatrial nodal artery. Histopathological examination of the mitral valve was consistent with IE and subsequently analyzed by universal polymerase chain reaction (PCR), which was positive for NG as the causative organism. The patient was given one dose of azithromycin and continued ceftriaxone to complete a six-week course. 

Additional sexual history obtained during the hospitalization revealed multiple sexual partners and a lack of routine sexually transmitted infection screening or consistent use of barrier protection. Gonococcal nucleic acid amplification testing was deferred due to prolonged antibiotic exposure. The patient's clinical course highlighted the aggressive and extensive nature of gonococcal IE and the diagnostic challenges with culture-negative presentations.

## Discussion

NG is an uncommon but highly virulent cause of IE. Unlike many bacterial pathogens, NG does not elicit a protective immune response; thus, individuals can be repeatedly infected, and persistent transmission may ensue within a population [8]. Additionally, NG has the capacity for antigenic variation to evade immune response [9]. Ceftriaxone continues to remain as the first-line therapy due to increased rates of resistance to fluoroquinolones [10].

Many times, a thorough sexual history is not included in initial patient interviews. This case highlights the importance of incorporating a thorough history, including sexual history, when a source of infection is not easily identified. This is especially important for less common causes of IE, such as NG. The diagnosis in this case met definite IE by the modified Duke criteria, supported by the histopathologic evidence of infection and the molecular identification of NG from the valvular tissue. In cases of culture-negative IE, the differential includes organisms such as *Haemophilus*, *Aggregatibacter*, *Cardiobacterium*, *Eikenella*, and *Kingella* (HACEK) species, *Coxiella burnetii*, and *Bartonella* species. Molecular diagnostics such as universal PCR can therefore be valuable for organism identification when conventional blood cultures fail. 

## Conclusions

Recognition of atypical infectious causes such as NG is important in patients presenting with aggressive or culture-negative IE, particularly when embolic phenomena are present. This case underscores the importance of obtaining a thorough sexual history which may facilitate timely recognition and management. Additionally, this case illustrates the importance of maintaining a broad differential diagnosis and utilizing adjunctive diagnostic modalities, such as PCR, to establish an accurate diagnosis to treat this potentially fatal condition.

## References

[1] Unemo M, Seifert HS, Hook EW 3rd, Hawkes S, Ndowa F, Dillon JR (2019). Gonorrhoea. Nat Rev Dis Primers.

[2] Tuddenham S, Hamill MM, Ghanem KG (2022). Diagnosis and treatment of sexually transmitted infections: a review. JAMA.

[3] Akkinepally S, Douglass E, Moreno A (2010). Tricuspid valve gonococcal endocarditis: fourth case report. Int J Infect Dis.

[4] Weiss PJ, Kennedy CA, McCann DF, Hill HE, Oldfield EC 3rd (1992). Fulminant endocarditis due to infection with penicillinase-producing Neisseria gonorrhoeae. Sex Transm Dis.

[5] Ramos A, García-Pavía P, Orden B (2014). Gonococcal endocarditis: a case report and review of the literature. Infection.

[6] Nie S, Wu Y, Huang L, Pincus D, Tang YW, Lu X (2014). Gonococcal endocarditis: a case report and literature review. Eur J Clin Microbiol Infect Dis.

[7] Staneck JL, Vincent S (1981). Inhibition of Neisseria gonorrhoeae by sodium polyanetholesulfonate. J Clin Microbiol.

[8] Shaughnessy J, Ram S, Rice PA (2019). Biology of the Gonococcus: disease and pathogenesis. Methods Mol Biol.

[9] Liu Y, Feinen B, Russell MW (2011). New concepts in immunity to Neisseria gonorrhoeae: innate responses and suppression of adaptive immunity favor the pathogen, not the host. Front Microbiol.

[10] Workowski KA, Bachmann LH, Chan PA (2021). Sexually transmitted infections treatment guidelines, 2021. MMWR Recomm Rep.

